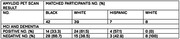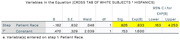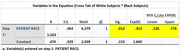# Differences in amyloid positivity rates among racial groups with cognitive impairment in a suburban‐rural Southeast community

**DOI:** 10.1002/alz.093824

**Published:** 2025-01-09

**Authors:** Eduardo Garcia Chacon, Annalise C Woodring, Monica K Crane

**Affiliations:** ^1^ The University of Tennessee‐Knoxville, Knoxville, TN USA; ^2^ Genesis Neuroscience Clinic LLC, Knoxville, TN USA

## Abstract

**Background:**

Underrepresented racial and ethnic groups have higher rates of clinical dementia symptoms and are given clinical diagnoses of Alzheimer’s disease without confirmatory AD biomarkers.

**Method:**

We compared amyloid PET positivity in cohort of all rural and suburban Southeastern participants from a single clinical practice who met appropriate‐use criteria for amyloid PET imaging between January 2020‐December 2024. We used a paired nominal date test McNemar test to compare amyloid PET positivity proportions between matched racial and ethnic groups and multivariable logistic regression to assess the odds of having a positive amyloid PET scan.

**Result:**

Data from 94 individuals (6 Asian/Pacific Islander(Other), 42 Black, 7 Hispanic, and 39 White) with MCI or dementia and amyloid PET were analyzed between April 2020 and December 2023. The median (range) age of participants was 75 (68‐82) years; 51 participants (54.3%) were female and 43 (45.7%) were male. For patient amyloid status, 49 participants (52.1%) had a validated negative amyloid result, and 45 participants (47.9%) had a validated positive amyloid result. In the optimal 1:1 matching analysis (n = 81, White participants had a greater proportion of positive amyloid PET scans analyzed but did not achieve statistical significance when compared with Black participants (24 of 39; 61.5%; 95% CI, 0.38‐0.72 vs 14 of 42; 33.3%; 95% CI, 0.19‐0.51, respectively) p>0.05. In the adjusted model, the odds of having a positive amyloid PET scan were lower for Black participants (OR, 0.313; 95% CI, 0.126‐0.776; P = 0.012), and Hispanic participants (OR, 0.33; 95% CI, 0.163‐4.253; P>0.05) compared with White participants.

**Conclusion:**

The proportion of amyloid positive PET scan was greater among White as compared with Black and Hispanic participants when controlling for social and demographic factors. These findings may reflect differences in underlying pathology and raises concern that other factors may be contributing to higher levels of cognitive impairment in underrepresented groups.